# Knowledge and Consumption Patterns of Omega-3 Fatty Acids Among the Central Balkan Population—A Prospective Cross-Sectional Study

**DOI:** 10.3390/nu17010122

**Published:** 2024-12-30

**Authors:** Maja Hitl, Nebojša Kladar, Jelena Banović Fuentes, Katarina Bijelić, Mirjana Đermanović, Ljilja Torović

**Affiliations:** 1 Department of Pharmacy, Faculty of Medicine, University of Novi Sad, 21000 Novi Sad, Serbia; maja.bekut@mf.uns.ac.rs (M.H.); nebojsa.kladar@mf.uns.ac.rs (N.K.); 990d15@mf.uns.ac.rs (J.B.F.); katarina.bijelic@mf.uns.ac.rs (K.B.); 2 Center for Medical and Pharmaceutical Investigations and Quality Control (CEMPhIC), Faculty of Medicine, University of Novi Sad, 21000 Novi Sad, Serbia; 3 Department of Bromatology, Faculty of Medicine, University of Banja Luka, 78000 Banja Luka, Bosnia and Herzegovina; mirjana.djermanovic@med.unibl.org; 4 Public Health Institute, 78000 Banja Luka, Bosnia and Herzegovina

**Keywords:** dietary supplements, diet, nutrients, questionnaire, public health

## Abstract

Omega-3 fatty acids (ω-3-FAs) represent a group of essential nutrients, but modern diets often do not allow for a balanced ratio between the intakes of ω-6-FA and ω-3-FA, which is vital for health. ω-3-FA can be found primarily in algae and fish, while the intake of ω-3-FA dietary supplements can be seen as an efficient way of providing nutrients important for many physiological functions. Background/Objectives: The aim of this research was to investigate the use of ω-3-FA-rich food and supplements, as well as the knowledge and attitudes on these nutrients among residents of the central Balkans—the Republic of Serbia and the Republic of Srpska. Methods: The research was performed as a prospective, cross-section, online survey. Results: A total of 895 responses were collected, with relatively high usage of ω-3-FA supplements (34.2%). It was found that the respondents use these supplements due to inadequate dietary intake, but also in therapy or prevention of certain diseases and conditions. Users take the supplements on a regular basis, although for short periods of time. The respondents reported the dietary intake of food rich in ω-3-FA. It was found that more than half of parents give these supplements to their children, with similar purposes, although more frequently and for longer periods of time. The use of ω-3-FA via supplements in pregnant and breastfeeding women is also present. Conclusions: The residents of the investigated territory seem to have an awareness of the importance of ω-3-FA use, with its consumption being registered in both the general population and specific subpopulations. Future steps would include further promotion and education on the given topic.

## 1. Introduction

Omega-3 fatty acids (ω-3-FAs) represent a group of long-chain polyunsaturated fatty acids with an important place in the homeostasis of human organisms. Among them, some of the most important are eicosapentaenoic acid (EPA) and docosahexaenoic acid (DHA). Besides them, an important precursor which can be converted to these biomolecules is alpha-linolenic acid (ALA) [1]. While ALA can be found in plants, primarily seeds, nuts, and vegetable oils, EPA and DHA are found in algae and fish, including the liver of lean, “white” fish (e.g., cod, halibut, etc.) and the entire body of fatty, “blue” fish (e.g., salmon, herring, tuna, etc.) [2]. Bearing in mind that these nutrients are essential, their adequate intake is the critical step which determines further benefits in human health [3]. The research supports dietary interventions to raise ω-3-FA levels and maintain a low ω-6-FA to ω-3-FA ratio to prevent early deaths from heart disease, cancer, or other causes [4]. An increase in ω-6-FA but also a decrease in ω-3-FA intake in the diets of modern Western societies, resulting in an ω-6/ω-3 ratio of 20:1 or higher, has contributed to the rising prevalence of hypertension, coronary heart disease, diabetes, obesity, autoimmune diseases, rheumatoid arthritis, and cancer [4,5,6].

The dietary reference values suggest that infants (>7 months) and children (up to 1 year old) require 100 mg of DHA per day, children from the age of 2 and adults require 250 mg of DHA + EPA per day, and pregnant and breastfeeding women require an additional 100–200 mg of DHA per day [7]. Unfortunately, modern diets are often depleted of many valuable nutrients and compounds with great benefits for the human organism, including ω-3-FA. A systematic review conducted in 2017 concluded that the intake of these nutrients was suboptimal even in specific subpopulations in European countries, including infants and children, as well as in the elderly (>65 years old) population [8]. Thus, the use of supplements containing ω-3-FA can be seen as a simple yet effective way to obtain the required nutrients.

The studies of Mogendi et al. [9] highlight four groups of key determinants of consumer understanding and evaluation of nutritious foods: “(1) nutrition knowledge and information; (2) attitudes, beliefs, perceptions and behavioural determinants; (3) price, process and product characteristics; and (4) socio-demographics”. According to these authors, “the modern consumer views their kitchen cabinet more and more as a medicine cabinet”. The numerous and diverse health benefits of ω-3-FA, proven in clinical settings, could really support such a view. These nutrients are seen as highly important for maintaining the health of the cardiovascular system, with this effect being achieved via several mechanisms, including positive effects on lipid metabolism and its vascular deposition [10,11]. The role of ω-3-FA in the normal functioning of the central nervous system is also frequently reported, with these fatty acids having both a structural and functional role in the brain; additionally, ω-3-FAs are considered as biomolecules affecting cognitive functions and preventing several degenerative diseases of the brain [12]. Positive effects of these nutrients are also reported on the cells of immune system [13], this being correlated with anti-inflammatory properties in pathological processes with pro-inflammatory mediators, as well as in autoimmune disorders [14]. The adequate intake of ω-3-FA is also vital in the specific population of pregnant and breastfeeding women, with fatty acids participating in the normal development of the brain and retina in utero, later resulting in the normal functioning of these organs [15]. It was also previously reported that supplementation with ω-3-FA can prevent early preterm and preterm births [15,16]. Regarding the role in breastfeeding, the adequate intake of ω-3-FA results in less frequent allergies in infants, potentially via the previously mentioned effects on immunity [15]. Numerous other beneficial roles in human health are still the subject of investigation. Importantly, a claim that declares, suggests, or implies a relationship between a food, or one of its ingredients and human health, a so-called health claim [17], when used on food/supplement labels and advertising, can be a very efficient means influencing consumers’ perceptions and beliefs, providing them with information and thereby improving their nutritional knowledge. The desired result—a positive influence on consumption behavior through helping consumers to make better-informed food choices—depends on their trust and understanding of such claims. In the case of ω-3-FA, numerous claims referring to the aforementioned health benefits of their consumption have been authorized in the European Union Register of nutrition and health claims [18]. A recent survey on regulatory compliance of health claims listed on ω-3-FA dietary supplements produced in more than 20 countries and marketed in the central Balkan region revealed a relatively high compliance rate of 86.8% among the evaluated supplements [19].

Previous studies suggest that the population of the central Balkans (Republic of Serbia and Republic of Srpska) is keen on using dietary supplements in the prevention of and therapy for gastrointestinal, hepatic [20], and cardiovascular [21] diseases and conditions, as well as in circumstances such as the COVID-19 pandemic [22]. Besides detecting the usage, it is important to investigate the patterns of use, as well as the attitudes of patients towards these supplements. Finally, it is also important to estimate the knowledge of patients on certain types of dietary supplements.

Bearing in mind the stated, the aim of this study was to investigate the use of ω-3-FA supplements, as well as to research the dietary habits related to the intake of ω-3-FA. The details on the usage, knowledge, and opinion on these fatty acids were investigated. Additionally, similar data were obtained in specific groups: toddlers and pregnant and breastfeeding women.

## 2. Materials and Methods

This research was performed as a prospective, cross-section study. The survey was conducted in a form of questionnaire, which the interviewees filled in by themselves. The questionnaire was divided into several sections: socio-demographic data, therapy with conventional drugs, dietary supplements containing ω-3-FA, diet, ω-3-FA in infants and toddlers, and ω-3-FA in pregnant and breastfeeding women (with a section on ω-3-FA in infants and toddlers being filled in by parents for their children and the last two intended only for female respondents). Some of the questions (primarily those on ω-3-FA supplements) were taken from a previously published survey [23]. In cases of respondents not using ω-3-FA supplements, a section investigating this matter was also created. All the questions were in the Serbian language, in Latin script; a questionnaire translated to the English language is given in the Supplementary Materials.

The survey was created using the Google Forms platform, and the corresponding link to the questionnaire was placed on the website of the Center for Medical and Pharmaceutical Investigation and Control Quality (CeMPhIC) of the Faculty of Medicine Novi Sad (https://cemphic.mf.uns.ac.rs/istrazivanje-omega-3-masne-kiseline/ (accessed on 31 October 2023)). The study was distributed via the Internet, using non-formal groups (e.g., social networks) among the population of the central Balkans—the Republic of Serbia and the Republic of Srpska. The study started in May 2023 and ended in October 2023.

The study was approved by Ethical Committee of Faculty of Medicine, University of Novi Sad, with the number of the approval document being 01-39/46/1. The respondents gave their consent for collecting personal data on the first page of the survey. Before the start of survey, a brief explanation of the study and its purpose and aims was given. Additionally, it was explained that participation is on a voluntary basis, that they can withdraw at any part of the survey, and that the anonymity of individuals is guaranteed.

A pilot survey was performed before the start of the research. A total of 25 respondents gave their answers, and feedback on the questionnaire was evaluated. After some technical changes, the survey was finalized. These 25 answers were excluded from further analysis.

After the end of investigation period, the data were summarized by the application of Microsoft Office Excel (v2019) in the form of a data matrix with dimensions of 896 × 154 and statistically analyzed using TIBCO Statistica v.13.5. Regarding the descriptive statistics, the results are presented as recorded frequencies (numbers and percentages). Questions regarding the opinions of respondents, which were answered using a Likert scale (with five degrees), were presented using Microsoft Excel v.2019. In cases where several answers were possible, the results are presented as number of reported uses (RU). Furthermore, in order to evaluate the potential patterns of recorded data (answers) variability, multiple correspondence analysis (MCA) has been applied. MCA, as a dimension reduction technique, is applied on qualitative variables and enables the representation of large datasets described by multiple variables in low-dimensional Euclidian space defined by the lower number of variables—correspondent axes—while, at the same time, preserving a certain share of the original dataset variability (inertia). By utilizing this approach, it is more convenient to identify specific patterns of the original dataset variability.

## 3. Results and Discussion

### 3.1. General Data on Respondents

In total, 895 responses to the survey were collected. The socio-demographic data of respondents are given in Table 1, while anthropometric features and other lifestyle data of importance are given in Table 2. The results are in accordance with previously conducted surveys investigating the use of fish oil/ω-3-FA in other geographical regions [23,24].

Interestingly, approximately three-quarters of respondents reported occasional or regular consumption of ethanol-containing beverages. It was previously shown that the excessive intake of ethanol leads to many and versatile harmful effects on human health, with the potential of ω-3-FA in alleviating some of the diseases induced by alcohol consumption being insufficiently investigated [25,26]. The present study also detected high consumption of caffeine products (nearly 90% of respondents are chronic or occasional consumers). To the best of our knowledge, there are no data on the effect of caffeine (an alkaloid from coffee and other beverages containing it, e.g., “energy drinks”) on the pharmacokinetics of ω-3-FA. Contrary to what has been previously said, some data on the correlation between ω-3-FA and nicotine (an alkaloid from tobacco) are available in the published literature. Interestingly, it was found that use of ω-3-FA supplements can positively affect “heavy smokers” by reducing the craving and the number of smoked cigarettes and also by reducing the oxidative stress induced by smoking [27]. Unfortunately, another previous study has found that smokers tend to have a lower intake of food rich in ω-3-FA [28], which is of particular importance since the present study shows that one-third of respondents are chronic or occasional consumers of cigarettes or other tobacco products.

### 3.2. Use of Conventional Drug Therapy

**Figure 1:**
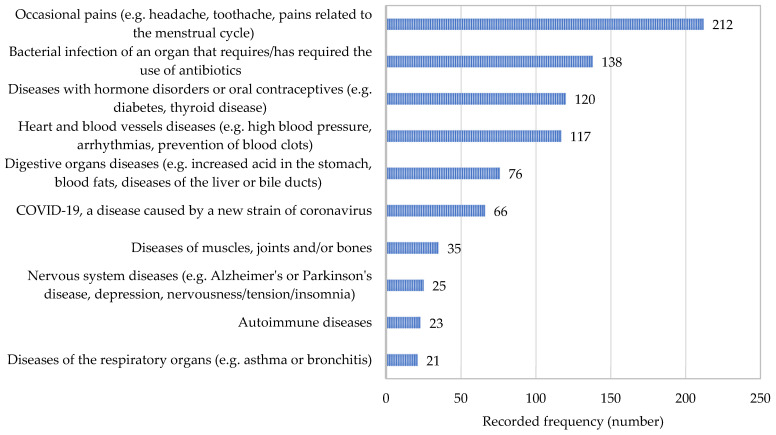
The most frequently reported reasons for the use of conventional drugs.

**Figure 2:**
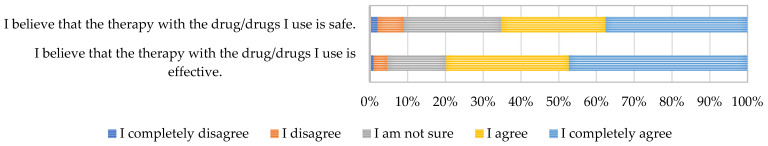
The reported opinions on the safety and efficacy of conventional drug therapy.

A total of 506 persons (56.5%) declared the use of conventional drugs for various conditions and diseases. The most frequently reported reasons for the use of conventional drugs, led by occasional pain, are listed in Figure 1. According to the results presented in Figure 2, the majority of respondents consider therapy with conventional drugs to be efficient and somewhat lower percentages (completely) agreed with the safety of these drugs.

### 3.3. ω-3-FA Dietary Supplements

Regarding supplements containing ω-3-FA, a total of 306 persons (34.2% of total number of respondents) declared using these supplements. Lower percentages of ω-3-FA users were recorded on the territory of New Zealand (21.9%) [24] and in the specific subpopulation of mid-aged (56–61 years-old) women in Australia (26.8%) [29].

In order to evaluate whether the respondents identified supplements containing ω-3-FA correctly, they were asked to name the brand of dietary supplement. In most of the cases, the respondents identified the supplements correctly, and some of them could not remember the name of the supplement/manufacturer.

**Figure 3:**
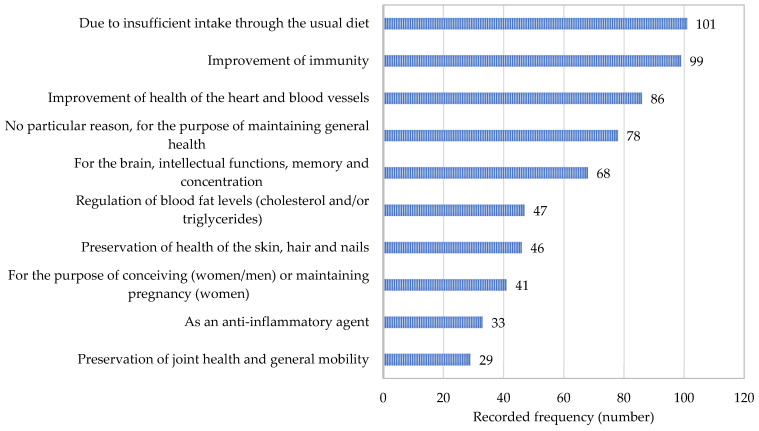
The most frequently reported reasons for the use of dietary supplements containing omega-3 fatty acids.

A list of the most frequently stated reasons for the use of supplements containing ω-3-FA is given in Figure 3. Insufficient intake through usual diet and the improvement of immunity were practically in equal lead, followed by heart health, general health, and brain health.

Some previous studies reported similar reasons for the use of ω-3-FA supplements. The top three reasons for use of fish oil supplements in New Zealand were general well-being (72.6%), the improvement of brain functioning (54.8%), and the treatment of joint pain and inflammation (31.5%) [24]. Achieving overall health and wellbeing was also the top reason (63.5%) for the use of ω-3-FA in three Eastern Asian countries, followed by “cognitive performance” (49.3%) and “support of nutritional intake” (45.3%) [23]. A specific subpopulation of patients with cardiovascular diseases in United States, with all respondents using ω-3-FA, reported that the main reason (34%) was general health, followed by the prevention and treatment of various cardiac and vascular diseases [30].

**Figure 4:**
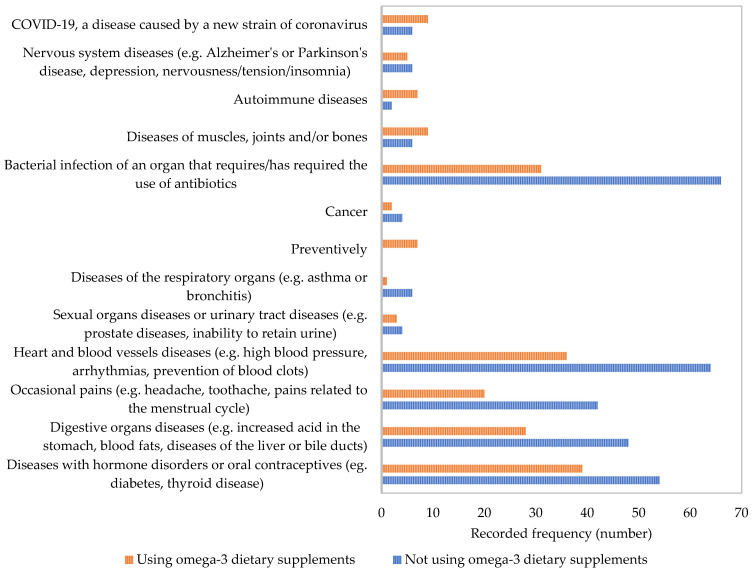
Respondents reporting simultaneous use or non-use of omega-3 fatty acid supplements and conventional drug therapy for various diseases and conditions.

Figure 4 contains the recorded frequencies of ω-3-FA supplement users also using conventional therapy for various diseases and conditions. Most frequently, users of ω-3-FA supplements simultaneously use conventional drugs for hormonal disorders and cardiovascular diseases as well as anti-infective drugs for bacterial infections. A previous study by [29] in Australian mid-age women found that women with osteo-arthritis and joint pain are more likely to use ω-3-FA. Regarding the use of supplements because of the COVID-19 pandemic, some researchers report the use of ω-3-FA for this reason. Their use was recorded in Saudi Arabia [31], but also in the Balkan peninsula [22].

When asked about the frequency of use, it was found that more than half of users (186, 60.8%) used ω-3-FA supplements on a daily basis. Approximately one-third of the respondents stated that they used these supplements two or more times a week (83, 27.1%). The rest of the respondents used ω-3-FA supplements less frequently (once a week 4.6%; once every two weeks 2.9%; once a month 4.6%). Similar proportions of ω-3-FA supplement users in New Zealand were previously recorded [24]. A study in cardiological patients in United States found that these patients are using fish oil supplements once (57%) or even twice a day (26%) [30].

Regarding the duration of supplementation, in most of the cases (47.1%), supplements containing ω-3-FA were used for less than three months, followed by supplementation lasting between three and six months (27.1%) or more than twelve months (16.3%). Similarly, ω-3-FA supplement users in Asian countries (these including China, Vietnam, and Thailand) reported their use for a median of four months [23]. These results are in contrast with a study conducted in New Zealand, where the majority of interviewees stated that supplementation lasted longer than 12 months [24].

The respondents also provided data on the dose of the used supplements. Approximately two-thirds of respondents (63.7%) reported that they use the dose suggested by the manufacturer. Further on, similar percentages of respondents reported that they use a dose suggested by the person who suggested the use of supplements in the first place (17.6%) or that they do not use any specific recommended dose (14.4%). Finally, some respondents reported that they opted for a dose which they found to be adequate in the literature (4.2%). The study conducted in New Zealand investigated doses in terms of milligrams of ω-3-FA source fish oil, with similar percentages of users in the following groups: 1000–1500 mg, 1500–2000 mg, and over 2000 mg [24]. Similarly, survey conducted in China, Vietnam, and Thailand estimated intake in milligrams of DHA, EPA, and total ω-3-FA, with 260, 60, and 320 mg per day, respectively [23].

In most of the cases, the decision on the use of ω-3-FA-based supplements was made by the respondents themselves (139, 45.4%). The advice of a health professional was the most important for 125 respondents (40.8%), this being between 17.8% of users following suggestions for ω-3-FA supplements in New Zealand [24] and 61.8% of respondents in Asian countries [23]. The third most frequent answer was following the advice of a family member, a partner, or a friend (11.4%), with this being similar to the portion of respondents from New Zealand using fish oil supplements (15.1%) [24]. Among patients with cardiovascular diseases in the United States, most frequently, the decision was made after a relative’s suggestion (32%) or their own decision (30%); regarding health professionals, most frequently, they followed the advice of a pharmacist, this being in high contrast with the present study [30].

Less than half of respondents (128, 41.8%) reported the use of ω-3-FA-based supplements to their doctor. The survey from New Zealand found that 62.8% of respondents inform their general physician on the use of supplements (these including but not limited to fish oil supplements) [24].

Most of the respondents bought their supplements at a pharmacy store (247 RU, corresponding to approximately three-quarters of all responses), followed by drug stores (shops with cosmetics, hygienic products, household chemicals, and some food and dietary supplements) (39 RU) and online shopping (25 RU). The study conducted in United States found different proportions of the locations of fish oil supplement purchase, with them being most frequently bought at grocery stores (30%), followed by mail order/online shopping (23%) and pharmacies (19%) [30].

When asked about the criteria for selecting supplements, similar numbers of respondents stated that they had bought the supplement that was suggested to them (114 RU) or supplements that they considered high-quality (130 RU). A substantially lower number of respondents chose the supplements based on the manufacturer (57 RU), while some made the choice based on the price of the supplement (38 RU).

**Figure 5:**
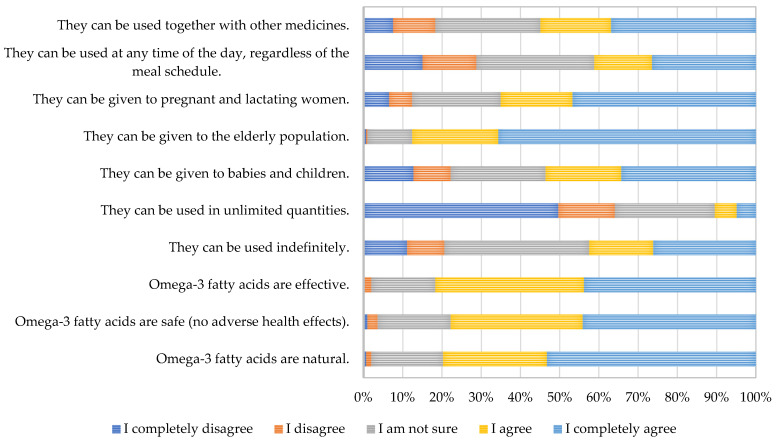
Respondents’ opinions and knowledge on the use of dietary supplements containing omega-3 fatty acids.

Regarding the opinion and knowledge on the use of dietary supplements, respondents were asked a series of questions; the results are presented in Figure 5.

The present study shows that a majority of respondents considered ω-3-FA as natural, safe (without adverse effects), and effective. In the study by [23], it was found that participants of the survey who perceived ω-3-FA supplements as proven by research to be safe and natural, were more likely to engage in their use. Also, the respondents of the present study seem to support their use in various subpopulations (infants and children, elderly population, and pregnant and breastfeeding women). Regarding other questions, most of the respondents consider that ω-3-FA cannot be used in unlimited quantities; however, nearly half of respondents consider that ω-3-FA can be used for extended periods of time. The presented findings indicate that public health professionals should work on the improvement of the knowledge of the general population on the adverse health effects of excessive amounts of ω-3-FA and their possible interactions with drugs and provide specific information regarding the proper way of usage and the amounts of omega-3-FA.

In order to test the knowledge of respondents, they were asked to write a name of a ω-3-FA (if they knew any). Slightly more than one-third (35.6%) of respondents knew to list ALA, EPA, and/or DHA; these results are somewhat higher than the 26.3% of cardiological patients form United States who knew to list the active ingredients in these supplements [27]. However, a significant proportion of respondents (16.7%) in the present study listed the name of the dietary supplement, as given by the manufacturer, instead of listing the ω-3-FA. When asked to name the source(s) from which it is possible to obtain ω-3-FA, if they knew any, most of the respondents listed fish as the primary source of ω-3-FA (228 answers), followed by nuts (52 answers) and linseed and linseed oil (42 answers).

**Figure 6:**
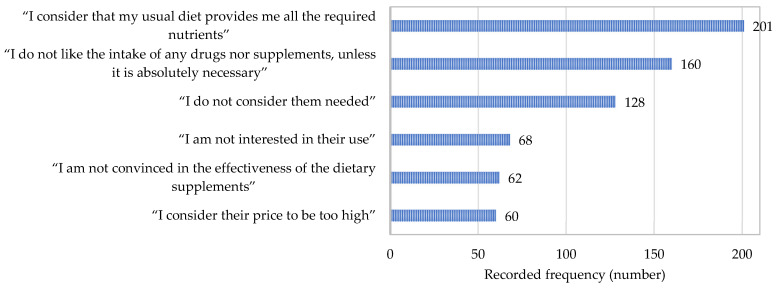
The most frequently reported reasons for not using dietary supplements containing omega-3 fatty acids.

A total of 65.8% of all respondents (589 answers) declared themselves as non-users of ω-3-FA supplements. The most frequently reported reasons for not using these supplements are presented in Figure 6.

Present study found that most of respondents not using ω-3-FA supplements consider that their diet provides all nutrients, including ω-3-FA. A high number of them expressed a lack of need and a lack of interest, and some of them found the price to be a limiting factor. Also, some of the respondents expressed an aversion for these supplements. The survey study conducted in China, Vietnam, and Thailand found that non-usage of ω-3-FA supplements is associated with limited accessibility and a lack of time and interest; however, in contrast to the present study, the cost of supplements was not found to be a factor associated with lower consumption [23]. On the other hand, the acceptance of fish among USA consumers was dependent on the cost [32].

**Figure 7:**
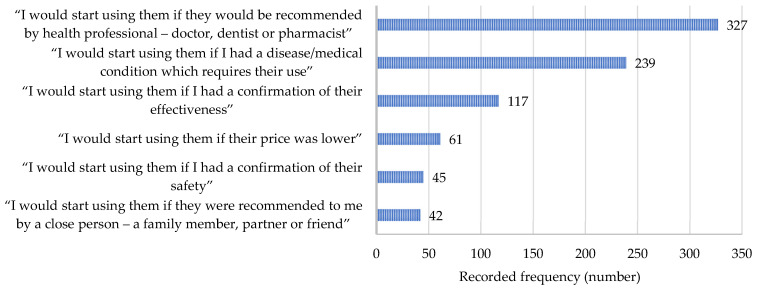
The most frequently reported reasons why respondents would consider using dietary supplements containing omega-3 fatty acids.

Additionally, the respondents were asked to state the case when they would reconsider and start the use of ω-3-FA supplements; the answers are given in Figure 7. After recommendation (by both medical professionals and close persons), the second most significant reason was the presence of a medical condition or disease which would require the use of ω-3-FA supplements—this reason has also previously been found to be a significant one in a survey conducted in Asian countries [23].

### 3.4. Diet and ω-3-FA

The respondents were asked some details about their dietary habits. Approximately a quarter of respondents (217, 24.3%) confirmed being on a special type of diet. These regimens included diets with increased amounts of vegetables and fruits and reduced amounts of meat (65 RU), diets without sugars (“keto-diet”) (54 RU), diets without gluten (42 RU), vegan or vegetarian diets (34 RU), and others.

The majority of respondents said that they eat fish (809, 90.4%). Approximately half of them eat both lean and fatty fish (435, 53.8%), approximately one-third only eat fatty fish (221, 27.3%), and the rest only eat lean fish (153, 18.9%). The frequency of fish intake by diet varies: 271 (33.5%) stated eating the fish once a week, 226 (27.9%) once every two weeks, 217 (26.8%) once a month, and 93 (11.5%) two or more times a week. Out of a total of 895 respondents, approximately one-third (276, 30.5%) declared the use of both fish and ω-3-FA supplements. Interestingly, it was previously found that persons who eat fish on a regular basis are, at the same time, more likely to use supplements containing ω-3-FA [23,24]. Regarding the consumption of other sea-food delicacies, less than half of respondents stated eating them (385, 43.0%). Among them, the most frequently consumed sea-food include squid (339 RU); crabs, shrimps, and lobsters (122 RU); various shellfish (121 RU); and octopuses (98 RU).

Respondents were asked about the use of other food considered to be an important source of ω-3-FA. They most frequently reported the use of various nut fruits (nuts, almonds, hazelnuts, etc.) (825 RU), linseed (403 RU), and chia seed (371 RU). Among dietary fats and oil, the respondents most frequently used sunflower oil (680 RU), olive oil (667 RU), and animal fats (480 RU). The majority of answers revealed that respondents do not use less common cooking oils (788 RU), and among those used, black cumin oil (52 RU), evening primrose oil (28 RU), and hemp oil (16 RU) were mentioned. A study on relative preferences for sources and food vehicles for long-chain ω-3-FA found that oilseeds rich in ω-3-FA, especially when incorporated in other food (e.g., bread, milk) are the preferable source as seen by USA consumers [32].

### 3.5. Multiple Correspondence Analysis—General Adults as Consumers of Omega-3 Fatty Acid Dietary Supplements

**Figure 8:**
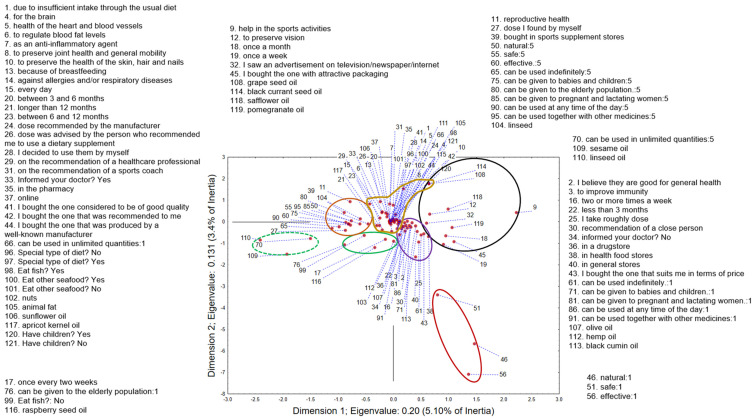
Multiple correspondence analysis—general adult consumers of omega-3 fatty acid dietary supplements. The position of the recorded answers in the space defined by the first two correspondent axes.

The application of MCA on the dataset containing the answers of respondents using ω-3-FA regarding their opinions on the safety and efficacy of ω-3-FA, reasons for using it, and details of application and dietary habits shows that the first two correspondent axes (Dimension 1 and Dimension 2) describe around 8.5% of original dataset variability. The position of the recorded answers in the space defined by the first two correspondent axes (Figure 8) indicates, in the positive part of the first axis and the negative part of the second axis, a group of respondents that, at first glance, deviates from the others (circled in red). These respondents do not agree with the fact that ω-3-FAs are natural, they do not believe that they are safe, and they consider them ineffective. The group located in the upper right quadrant (circled in black) included respondents who purchased ω-3-FA supplements based on attractive packaging or advertising. They do not follow the recommendations available on the product labeling and take supplements once a week/once a month to help with sports activities or to improve their eyesight. Also, they use some specific oils in their diet, such as grape seed oil, black currant seed oil, safflower oil, and pomegranate oil. The group located to the right of the intersection of the two axes, in the positive part of the first axis (circled in purple), included respondents who did not understand the significance of reporting ω-3-FA supplement use to the doctors, and usually consume them on the recommendation of a close person. They buy them in stores that are not specialized in the sale of these products (general store, health food stores), they do not take the recommended or any specified dose, and the price is a decisive factor when buying these supplements. They use the supplements two or more times a week, for a period shorter than three months, which may be related to the belief that they are good for general health and to improve immunity. Consumers from this group share the opinion that these supplements cannot be given to infants, children, pregnant women, and nursing mothers, which confirms that they do not have sufficient information about these products. They also share the opinion that ω-3-FA supplements cannot be combined with other medications and cannot be used at not-specified times of the day. When it comes to eating habits, in addition to olive oil, they also use some more specific oils, such as hemp oil and black cumin oil, which are often advertised. The group that included the largest number of respondents is located in the intersection of the two axes (circled in yellow). Therefore, the reasons for using ω-3-FA supplements are numerous: due to insufficient dietary intake; to improve cognitive function; to improve the health of the cardiovascular system; to lower blood fat levels; to improve the health of joints, skin, hair, and nails; against allergies and respiratory diseases; as an anti-inflammatory agent; and due to breastfeeding. Respondents from this group were responsible in taking these supplements, i.e., they take them every day for a long period of time (from 3 months to more than 12 months). They take these supplements on the recommendation of a health care professional, sports trainer or at their own discretion, in the dose recommended by the manufacturer or by the person who recommended them to take these products. Moreover, they think that ω-3-FA supplements should not be used in unlimited quantities, they have informed their doctor about their use and buy them in a pharmacy or online. They also buy those they know are of good quality, manufactured by well-known manufacturers, or those recommended to them by a health care professional. This group included respondents who were on a specific diet and those who were not, so no significant conclusion can be drawn. However, what they had in common was that they used fish, nuts, sunflower oil, and apricot kernel oil in their diet, while, when it comes to sea-food consumption, the answers were mixed. The group that was in the negative part of both the first and second axes (circled in green) was distinguished by the fact that they took supplements once every two weeks, did not eat fish, used raspberry seed oil, and shared the opinion that ω-3-FAs are not required to be given to the elderly population. The group that was in the negative part of the first axis and, for the most part, in the positive part of the second axis (circled in orange) included respondents who thought that ω-3-FAs are completely natural, safe, and effective and can therefore be given to infants, children, and pregnant or breastfeeding women, as well as to the elderly population. The dose they take is self-determined, which may be in accordance with their thinking that these supplements can be taken in unlimited amounts. Most often, they take supplements to improve reproductive health, buy them in sports supplement stores, fully agree that these supplements can be used at any time of the day, and that they can be combined with other drugs. When it comes to nutrition, the oil that is common to this group is linseed oil. The group that was in the negative part of both the first and second axis (green dashed circle) shares the opinions of the previously mentioned one but highlights the usage of sesame oil in the diet.

### 3.6. ω-3-FA and Infants and Children

The respondents were asked whether they have children and, if yes, how many of them. More than half of the respondents were parents of children (502, 56.1%), most frequently having 2 children (266, 53.0%) or 1 child (178, 35.5%) (mean 1.77, median 2, range 1–5). Out of all parents, approximately one-third (145, 28.9%) were parents of infants or toddlers.

**Figure 9:**
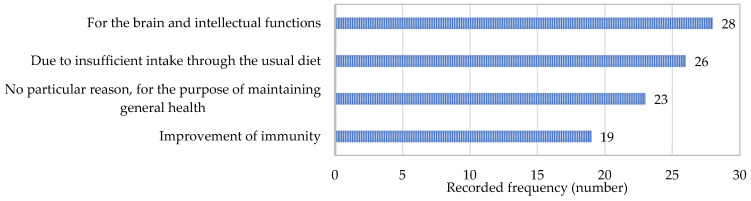
The most frequently reported reasons for use of dietary supplements containing omega-3 fatty acids in infants and toddlers.

Out of parents with infants and toddlers, approximately half of them stated giving their children a dietary supplement containing ω-3-FA (78, 53.8%). This percent is somewhat higher than in the study conducted in Eastern Asian countries, where 35% of parents stated giving ω-3-FA supplements to children, with a difference in the age of children, ranging between 1 and 18 years old [33]. As in cases with adults, parents were asked to give the name of the dietary supplement. In most of the cases, the parents listed dietary supplements with ω-3-FA, some of them listed multicomponent preparations (with ω-3-FA included), and some reported that they could not remember the name of the supplement. The list of reported reasons for giving children these supplements, given in Figure 9, shows a practically uniform distribution of responses between reasons related to specific functions and some general reasons. Similar results were obtained in the survey conducted in China, Thailand, and Vietnam, where the majority of parents stated giving these supplements with the purposes of “supporting growth/development”, “supporting cognitive performance”, and “supporting immune health” [33].

In most of the cases, parents gave ω-3-FA supplements to infants and toddlers every day (64, 82.0%), whereas others gave them two or more times a week (13, 16.7%). Additionally, most of the parents gave ω-3-FA supplements on a regular basis (continuously) (51, 65.4%), while a third of responding parents have been giving the supplements for less than three months (27, 34.6%).

Regarding the dose of ω-3-FA, the majority stated that they are giving the dose recommended by the manufacturer, given on the package of the product (46, 59.0%), followed by the dose advised by the person recommending the use of these ω-3-FA supplements (31, 39.7%). Favorably, in most of the cases, the use of ω-3-FA in children was advised by a health professional (57.7%) or through specific advice by a child’s pediatrician (34.6%). It is encouraging that in most of the cases (91.0%), a child pediatrician was informed on the use of ω-3-FA supplements.

**Figure 10:**
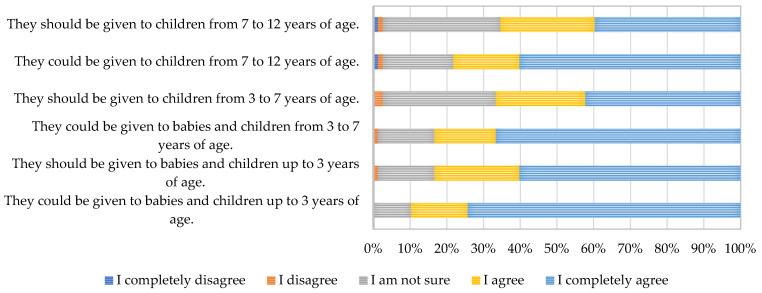
Reported opinions of parents on the use of omega-3 fatty acid dietary supplements in infants and children.

All the parents giving supplements to children were asked for their opinion, regarding the use of ω-3-FA supplements in children of various age. The results are present in Figure 10.

Parents were of the opinion that ω-3-FA supplements could and should be given to children of all age groups. However, a not-negligible number of parents was not sure about the answer, with an upward trend observed in connection with children’s age, reaching up to one-third of parents. Along with a low proportion of disagreeing parents, this suggests a need for engagement from health professionals in educational programs related to the nutritional importance and sources of ω-3-FA.

### 3.7. Diet and ω-3-FA in Infants and Children

The parents were asked whether their children were on some sort of special diet. Only five parents responded positively, with five answers stating their diet was adapted to an intolerance of lactose and two parents reporting intolerance to gluten in their children (with two children having intolerance both to lactose and gluten). Some previous studies suggest that the use of ω-3-FA supplements is more frequent in children with some health issues [33].

Approximately two-thirds of parents of toddlers responded that their children eat fish (101, 69.7%). The most of the parents stated that their children eat both fatty and non-fatty fish (53.5%), followed by 25.7% stating the use of lean fish only. Most frequently, children eat fish once a week (38.6%), followed by 30.7% eating fish once every two weeks. It was found that 9.8% of infants/toddlers are simultaneously consumers of fish products and ω-3-FA supplements. Interestingly, it was previously found that parents who give their children fish as part of a regular diet are more likely to also give ω-3-FA supplements [33].

When asked whether their children are eating other types of food known as good sources of ω-3-FA, in most of the cases, parents stated that they are giving their children various nuts (almonds, walnuts, hazelnuts…) (89 RU), linseed (34 RU), and chia seed (25 RU); however, a significant part of parents stated that they are not giving their children any of these foods (52 RU). Among dietary oils and fats, parents are most frequently giving olive oil (107 RU), sunflower oil (79 RU), and animal fats (71 RU).

### 3.8. Multiple Correspondence Analysis—Children as Consumers of Omega-3 Fatty Acid Dietary Supplements

**Figure 11:**
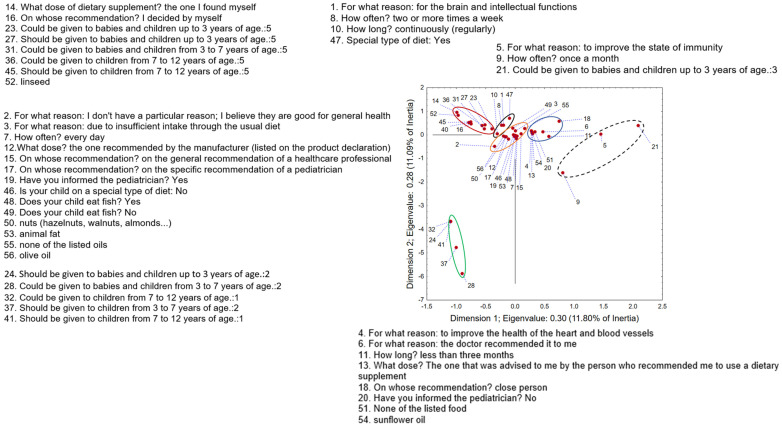
Multiple correspondence analysis—parents giving omega-3 fatty acid dietary supplements to their children. The position of the recorded answers in the space defined by the first two correspondent axes.

The application of MCA on answers given by parents which give ω-3-FA supplements to their children shows that the first two correspondent axes describe around 23% of original dataset variability. The position of the recorded answers in the space defined by the first two correspondent axes (Figure 11) indicates the group that stands out from the rest in the negative part of both the first and second correspondent axes (circled in green). Parents from this group share the opinion that ω-3-FA supplements should not/could not be given to infants and children of any age. In contrast to them, the group located in the negative part of the first and the positive part of the second axis (circled in red) includes those parents who think that these supplements should/could be given to infants and children of all ages, whose decision about taking and providing the dose of these supplements was made by parents, and whose diet includes the use of linseed. The group close to the previously mentioned one (circled in black) consisted of respondent-parents who give supplements based on ω-3-FA to their children for brain development and to improve intellectual functions; therefore, in order to manifest these effects, they give them to children two or more times a week and for a long period of time. Parents from this group probably have children with some health issues, because their children are on a special type of diet. All these components indicate that parents from this group are aware of the necessity of using ω-3-FA in their children’s diet. It is typical for parents from the group located at the intersection of the two axes (circled in orange) to think that ω-3-FAs are deficient in their regular diet and that they are good for general health and to therefore give their children supplements every day. It is recorded that parents give their children the doses recommended by the manufacturer, or by healthcare professionals, which indicates their health awareness. This is confirmed by the fact that they informed their pediatrician about the use of these supplements. Also, parents from this group do not have any specific dietary habits and give their children well-known foods that are rich in ω-3-FA (nuts, olive oil, fish). The group located in the positive part of both the first and second axes (circled in blue) included those parents who share the opinion that ω-3-FA supplements are good for the cardiovascular system, give them to children for less than three months, have decided to use these supplements (and selected the dose) based on the recommendation of a close person, and did not inform their pediatrician about the use of supplements. In their children’s diet, they do not use any of the specific foods rich in ω-3-FA, and they use oil that is most often used for food preparation in the investigated area: sunflower oil. The group of parents that was quite different from the others was located in the positive part of the first and the negative part of the second axis (circled in black dash). They give their children these supplements to improve immunity, so they use them periodically. They are also neutral when it comes to the justification of giving these supplements to infants and toddlers.

### 3.9. Diet and ω-3-FA in Pregnant and Breastfeeding Women

Among a total of 700 female respondents, 31 (4.4%) declared themselves as pregnant women. Among them, 9 were in the first trimester, 10 were in the second trimester, and 12 were in the third trimester. Nearly half of pregnant women declared the use of ω-3-FA supplements (45.2%). The majority of them stated that pregnancy is the single or one of the most important reasons for the use of these supplements (21.4 and 64.3%, respectively). Also, when they were asked whether the use of these supplements can be attributed to the suggestion of their doctor (gynecologist), four said yes, and four said that it is one of the main reasons, while six said no. It is also interesting to point out the view of some physicians regarding ω-3-FA: gynecologists in Germany consider ω-3-FA supplements less important in comparison to mineral and vitamin supplements. Additionally, their opinion is that supplementation is more important during the period of pregnancy than in the preconception period [34].

**Figure 12:**
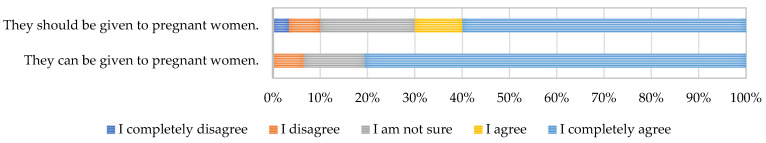
Reported opinions of pregnant women on the use of omega-3 fatty acid supplements.

All pregnant women were also asked their opinion on the use of supplements containing ω-3-FA (Figure 12). The proportion of those who do not agree or are not sure about the convenience and need of taking ω-3-FA supplements (around 20 and 30%, respectively) is worrying and urges the attention of health professionals.

**Figure 13:**
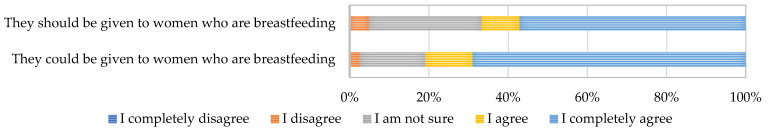
Reported opinions of breastfeeding women on the use of omega-3 fatty acid supplements.

Regarding women in the lactating period, 42 (6.0%) respondents declared themselves to be currently breastfeeding. On average, at the time of survey, women were breastfeeding for approximately 8 months (mean 8.1, median 7, range 0-24 months). Among them, less than half (41.6%) were using ω-3-FA supplements. The same number of respondents stated that lactation is not the reason for the use of these supplements and that it is one of the most important reasons (nine each), while two women stated that breastfeeding was the single most important reason. Also, the lactating women were asked whether the use of these supplements can be attributed to the suggestion of their doctor (gynecologist) or visiting nurse, and the majority stated that this is not the case (twelve), six said that it is one of the main reasons, and only two women said yes. Breastfeeding women in most of the cases expressed an opinion that they could and should use the ω-3-FA supplements (Figure 13). The proportion of those who deny or are not sure about the convenience/need of taking ω-3-FA supplements (around one-fifth and one-third, respectively) is very similar to the one observed among pregnant women.

### 3.10. Overall Considerations and Implications

The present study found that approximately two-thirds of respondents do not use supplements containing ω-3-FA. Several reasons were listed for the aforementioned behavior, including the belief that diet is being sufficient in providing all the required nutrients. However, contemporary studies have found that this is usually not the case, and ω-3-FA intake is commonly deficient in various subpopulations. The justification for respondents’ claims could be associated with the belief that ω-3-FAs are being consumed in sufficient amounts in cases of fish-enriched diets. However, the present study found that, although the proportion of people reporting the consumption of fish is quite high, the frequency of intake cannot be classified as satisfactory. Although high consumption of food rich in ALA was recorded, the conversion of ALA to bioactive ω-3-FA is relatively limited and should not be considered as a primary recommendation for ω-3-FA intake. The second most frequently reported reason for not using ω-3-FA supplements indicated that respondents would reconsider their use if they had a disease/condition requiring ω-3-FA intake. It must be stated that, besides the positive effects recorded due their intake in different pathological conditions, the consumption of ω-3-FA is also beneficial for the improvement of numerous physiological functions, thus suggesting that such information should be more promoted among the general population, as it is currently done with some other food components, e.g., fibers, vitamins, and minerals. It can also be pointed out that some respondents were not even interested in ω-3-FA-containing supplements; thus, they represent a group which was not even potentially informed on the benefits of ω-3-FA use in the first place. The present study also evaluated some aspects of self-perception of (healthy) lifestyle. And, while the intake of dietary supplements containing ω-3-FA can be evaluated as high, it must be highlighted that some data (from Table 2) suggest certain lifestyle parameters opposed to generally perceived healthy habits. This could potentially result in inadequate ω-3-FA intake due to a self-perceived healthy lifestyle and the inadequately estimated intake of these nutrients via food.

Regarding the respondents consuming ω-3-FA (both via food and dietary supplements), several aspects should be considered, which are related to the complexity of potential food–drug interactions. Namely, the present study reports a high frequency of conventional drug usage, with a significant proportion of respondents using drugs for the treatment and prevention of cardiovascular system disorders. This is of particular importance since these drugs are reported to have potential interactions with supplements containing ω-3-FA. Thus, health professionals (both, doctors prescribing drugs and pharmacists dispensing them) should take preventive measures in order to minimize the risk of potential interactions leading to adverse effects. Again, bearing in mind that ω-3-FA-containing supplements are not necessarily bought at pharmacy stores, and that some consumers may proceed with their use without the advice of a health professional, suggests that these supplements should be promoted by appropriate public health campaigns. The provided information should include recommendations on the adequate intake of ω-3-FA supplements—after a meal, possibly one containing higher amounts of dietary oils and fats—with the aim of better ω-3-FA absorption. Another important information is certainly the desired low ratio between intakes of ω-6-FA and ω-3-FA, which is vital for human health [4]. However, the maintenance of such a ratio is a rather complex issue, considering the rich content of ω-6-FA in plant oils that are usually used in the diets of the respondents.

With regard to the amounts of ω-3-FA consumed in the form of supplements, it is of interest to highlight that, expectedly, the majority of the respondents (63.7%) reported that they use the dose suggested by the supplement manufacturer, whereas only 4.2% of them opted for a dose which they found in the literature to be adequate. The majority of parents (59.0%) also stated that they give the dose recommended on the package of the product. However, a recent assessment conducted on 59 supplements purchased in the central Balkan region, all bearing authorized health claims referring to ω-3-FA, totaling 107 individual claims, revealed that the intake of ω-3-FA (calculated based on the labelled content of ω-3-FA per dosage unit and the recommended number of dosage units per day), when compared with the intake requirements defined for foods with ω-3-FA health claims [18], supported 85.0% of the claims and deemed 4.7% as unsubstantiated, while for the remaining ones, there were no sufficient data [19]. The same study showed that the contribution of fish oil supplements to the recommended daily intake of EPA/DHA ranged from 23% to 600% (average 216 ± 126%) for adults and from 120% to 600% (average 310 ± 132%) for children, while, in the case of pregnant and lactating women, as much as 70% of the supplements had no capacity to provide the recommended daily dose of EPA/DHA when consumed in the recommended amounts [19].

The current survey also aimed at investigating ω-3-FA use in specific subpopulations, such as infants and toddlers, as well as pregnant and lactating women. According to the recorded results, it can be concluded that there is awareness about the significance of providing appropriate ω-3-FA intake, especially by the population of children and individuals in specific physiological conditions. What seems to be the source of variability in ω-3-FA supplement intake is a lack of uniform recommendations provided by gynecologists, pediatricians, and visiting nurses to corresponding subpopulations (especially bearing in mind that the survey is formally conducted in two different states of the Balkan peninsula). Additionally, real-life results suggest that supplementation may start as early as from the first day of discharge from hospital delivery, while formal guidelines give data on supplementation from the third month of life. This suggests that doctors should provide advice on the importance of ω-3-FA supplement use to all delivering mothers, together with guidelines on proper application. An aspect this survey has not investigated in detail was the type of nutrition in infants—whether they were breastfed or were given infant formula. Namely, it can be considered that breastfeeding provides all the necessary nutrients, including ω-3-FA, if the intake of ω-3-FA in lactating mothers is adequate. Secondly, infants being fed exclusively by infant formula will depend on the content of ω-3-FA present in such products. Infant formulas represent one of the highly regulated food categories, with the aim of providing safe and adequate nutrition for one of the most vulnerable categories of the general population. However, taking into account the presence of infant formula products enriched with DHA, as well as those not containing DHA (DHA is the ω-3-FA specifically recommended for 7–11-month infants and 1-year-old children), on the market, of paramount importance is that attending doctors provide this information to new mothers who do not breastfeed.

### 3.11. Study Strengths and Limitations

The present study has several limitations. It was limited to literate respondents speaking the Serbian language and having no communication disabilities. With the survey being distributed online, the study was limited to those with Internet access (via computers or mobile phones). The survey relied on self-reporting data; thus, memory bias cannot be excluded. Although anonymity was guaranteed, the risk of misreporting the data remains present. Also, the potential of misidentifying supplements containing ω-3-FA existed (which was potentially excluded by asking the respondents about the brand and the names of fatty acids). On the other hand, there is also a risk of people using ω-3-FA supplements being more prone to filling in this survey, having a self-perception of knowing more on the given subject. Indeed, about 45% of respondents are in the medical/healthcare field, which likely contributes to the positive bias of the study’s findings.

However, it is also important to list the strengths of the present study. The survey was distributed to a relatively large and diverse group of respondents, covering a relatively large territory of two countries at the same time. The study investigated not only supplements containing ω-3-FA but also the dietary aspects of these nutrients. Additionally, the use of ω-3-FA was investigated in specific population subgroups (children, and pregnant and breastfeeding women) which were of particular interest for public health considerations.

Finally, questions arising from the current study, such as “Do these findings have clinical implications for the health of the population of the central Balkans?” and “Which are of utmost importance for public health authorities?”, need to be considered in future investigations.

## 4. Conclusions

The present study provides data on habits, knowledge, and opinions related to the usage of dietary supplements containing omega-3 fatty acids in populations of the central Balkans—the Republic of Serbia and the Republic of Srpska. The study findings indicate a relatively high proportion of the population using these supplements and satisfactory knowledge on the given topic. However, the gaps in knowledge and the deficit of uniform recommendations on the use of these supplements presented by health professionals in the countries covered by this study, as well as data on their use in specific populations, suggest that both the further education of the general population and forming a consensus opinion by health professionals regarding recommendations of omega-3 fatty acid use are required. These can be achieved through the better cooperation of all interested parties and appropriate public health authority-run educative courses and campaigns.

## Figures and Tables

**Table 1:** Socio-demographic data of respondents.

Question	Answers	Recorded Frequencies (Number and Percent)
gender	male	195 (21.8%)
	female	700 (78.2%)
age	˂20 years	17 (1.9%)
	21–35 years	395 (44.1%)
	36–50 years	333 (37.2%)
	51–65 years	137 (15.3%)
	>65 years	13 (1.5%)
highest level of education	no formal education	0 (0%)
	elementary school	6 (0.6%)
	high school	218 (24.4%)
	college	59 (6.6%)
	university	612 (68.4%)
type of education ^1^	high school student in medical field	20 (2.2%)
	college or university student in medical field	71 (7.9%)
	medical doctor, pharmacist, dentist, or other professional in medical field	318 (35.5%)
	none of the previous	486 (54.4%)
employment status	pupil or student	88 (9.8%)
	unemployed	61 (6.8%)
	employed	726 (81.2%)
	retired	20 (2.2%)
monthly income ^2^	˂EUR 300	132 (14.8%)
	EUR 300–600	170 (19.0%)
	EUR 600–900	283 (31.6%)
	>EUR 900	310 (34.6%)
region of residency	Republic of Serbia	494 (55.2%)
	Republic of Srpska	362 (40.4%)
	other	39 (4.4%)
type of settlement	urban	706 (78.9%)
	suburban	135 (15.1%)
	rural	54 (6.0%)
marital status	single	279 (31.2%)
	married	488 (54.5%)
	in extra-marital union	75 (8.4%)
	divorced	42 (4.7%)
	widowed	11 (1.2%)

^1^ It should be noted that around 45% of respondents are in the medical/healthcare field and, thus, probably more prone to filling in this survey because of the belief that they have sufficient prior knowledge of the topic. ^2^ Monthly income was converted to EUR from local currencies (RSD in the Republic of Serbia and BAM in the Republic of Srpska), according to the currency exchange rate at the time of conducting the research.

**Table 2:** Anthropometric features and other lifestyle data of importance.

Question	Answers	Recorded Frequencies (Number and Percent)
body mass index	˂18.5 (underweight)	33 (3.7%)
	18.5–24.9 (normal range)	515 (57.8%)
	25–29.9 (overweight)	261 (29.3%)
	≥30.0 (obese)	82 (9.2%)
alcohol consumption	no, never	217 (24.3%)
	yes, sometimes	648 (72.4%)
	yes, regularly	30 (3.3%)
nicotine consumption	no, never	596 (66.6%)
	yes, sometimes	150 (16.8%)
	yes, regularly	149 (16.6%)
caffein consumption	no, never	106 (11.8%)
	yes, sometimes	256 (28.6%)
	yes, regularly	533 (59.6%)
level of average physical activity	weak	245 (27.4%)
	average	254 (28.4%)
	moderate	324 (36.2%)
	intensive	72 (8.0%)
general satisfaction with respondent’s life quality	bad	28 (3.1%)
	average	432 (48.3%)
	very good	351 (39.2%)
	excellent	84 (9.4%)

## Data Availability

The original contributions presented in this study are included in the article/Supplementary Materials. Further inquiries can be directed to the corresponding author.

## Supplementary Materials

The following supporting information can be downloaded at: https://www.mdpi.com/article/10.3390/nu17010122/s1, Questionnaire.
