# Supplementary material for: Knowledge and Consumption Patterns of Omega-3 Fatty Acids Among the Central Balkan Population—A Prospective Cross-Sectional Study
Source: Nutrients. 2024 Dec 30;17(1):122. doi: 10.3390/nu17010122 (PMC11723183; doi:10.3390/nu17010122)
Supplement: Supplementary file 1 [file nutrients-17-00122-s001.zip › nutrients-3396924-supplementary.pdf]

Esteemed,

By participating in the survey called

"USE, KNOWLEDGE AND ATTITUDES ABOUT OMEGA-3 FATTY ACID DIETARY SUPPLEMENTS"

you will be part of a research that investigates the frequency of use of these nutritional supplements, awareness of the health risks and benefits of their use, as well as your personal attitudes and knowledge about them.

This research concerns your use of supplements based on omega-3 fatty acids, which include various encapsulated forms, drops, oils (eg fish oil, evening primrose oil, etc.), as well as dietary habits related to the intake of these fatty acids (olive oil, hemp oil, frequency of fish and seafood consumption...)

The questions in the questionnaire refer to application **currently or within the last year** (unless otherwise stated).

Your data is anonymous.

Participation in the survey is voluntary. It will take you about 15 minutes to fill out the questionnaire.

By filling out this questionnaire, you give your consent for us to process your data for scientific purposes.

The research is conducted by the Center for Medical-Pharmaceutical Research and Quality Control (CEMFIK), Faculty of Medicine, University of Novi Sad.

If you have additional questions or want additional information about this research, you can contact Maja Hitl, PhD, Teaching Assistant, via email address: maja.bekut@mf.uns.ac.rs

Thank you for your time!

SOCIODEMOGRAPHIC CHARACTERISTICS

Gender:

1. Male
2. Female

Age:

1. under 20 years
2. between 21 and 35 years
3. between 36 and 50 years
4. between 51 and 65 years
5. more than 65 years

Highest completed level of education:

1. No education
2. Elementary school
3. High school
4. College
5. University

According to the highest completed level of education, you are:

1. high school student in medical field
2. college or university student in medical field
3. medical doctor, pharmacist, dentist, or other professional in medical field
4. none of the above

Employment Status:

1. pupil or student
2. unemployed
3. employed
4. retired

Monthly income:

Republic of Serbia

1. under 36,000 dinars (<300€)
2. 36,000-72,000 dinars (300-600€)
3. 72,000-108,000 dinars (600-900€)
4. above 108,000 dinars (>900€)

Republic of Srpska

1. under 600 KM (<300€)
2. 600-1200 KM (300-600€)
3. 1200-1800 KM (600-900€)
4. above 1800 KM (>900€)

The region you live in:

1. Vojvodina
2. Belgrade region
3. Šumadija and Western Serbia
4. Eastern and Southern Serbia
5. Kosovo and Metohija
6. outside Serbia, on the territory of Europe
7. outside of Serbia, on the territory of America
8. Banja Luka region
9. Doboj-Bijelina region
10. Sarajevo-Zvornik region
11. Trebinje-Foča region
12. Brcko district
13. Federation of Bosnia and Herzegovina
14. outside Bosnia and Herzegovina, on the territory of Europe
15. other, specify:____________

Type of settlement where you live:

1. urban
2. suburban
3. rural

Marital status:

1. unmarried
2. married
3. in an extramarital union
4. divorced
5. widower/widow

Your body height: _____________cm

Your body weight: ______________kg

Do you consume alcohol?

1. No, never
2. Yes, sometimes
3. Yes, regularly

Do you smoke cigarettes?

1. No, never
2. Yes, sometimes
3. Yes, regularly

Do you consume coffee and other caffeinated beverages (e.g. energy drinks)?

1. No, never
2. Yes, sometimes
3. Yes, regularly

Rate your average (physical) activity level:

1. weak
2. satisfactory
3. moderate
4. intensive

How do you rate the overall quality of your life:

1. bad
2. average
3. very good
4. excellent

CONVENTIONAL DRUG THERAPY

Do you use/have you used any medicine (prescribed by a health professional - doctor, dentist, pharmacist):

YES NO

State the reason for using the medicine/medicines (*it is possible to circle more than one answer*):

1. digestive organs diseases (e.g. increased acid in the stomach, blood fats, diseases of the liver or bile ducts...)
2. heart and blood vessels diseases (e.g. high blood pressure, heart irregularities (arrhythmias), protection of blood vessels or prevention of blood clots...)
3. sexual organs diseases or urinary tract diseases (e.g. prostate diseases, inability to retain urine...)
4. diseases with hormone disorders or oral contraceptives (eg diabetes, thyroid disease...)
5. bacterial infection of an organ that requires/has required the use of antibiotics
6. COVID-19, a disease caused by a new strain of coronavirus
7. cancer
8. autoimmune diseases
9. diseases of muscles, joints and/or bones
10. nervous system diseases (e.g. Alzheimer's or Parkinson's disease, depression, nervousness/tension/insomnia...)
11. diseases of the respiratory organs (e.g. asthma or bronchitis...)
12. occasional pains (e.g. headache, toothache, pains related to the menstrual cycle...)
13. skin diseases
14. preventively
15. other, please specify:____________

On a scale of 1 to 5, rate how much you agree with the following statements:

	I completely disagree	I disagree	I am not sure	I agree	I completely agree
I believe that the therapy with the drug/drugs I use is effective.	1	2	3	4	5
I believe that the therapy with the drug/drugs I use is safe.	1	2	3	4	5

OMEGA-3 FATTY ACIDS

Do you use/have you used any dietary supplement based on omega-3 fatty acids?

YES NO

IF THE ANSWER IS YES

usage

Please provide the name(s) of the omega-3 fatty acid supplements you are using:

____

What is (was) the reason for using a dietary supplement based on omega-3 fatty acids (*it is possible to circle more than one answer*):

1. I don't have a particular reason, I believe they are good for general health
2. due to insufficient intake through the usual diet
3. to improve the health of the heart and blood vessels
4. for the brain, intellectual functions, memory and concentration
5. to improve immunity
6. against allergies and/or respiratory diseases
7. to preserve joint health and general mobility
8. to preserve vision and eye health
9. as a help in the sports activities I am engaged in
10. as an anti-inflammatory agent
11. to regulate blood fat levels (cholesterol and/or triglycerides)
12. to preserve the health of the skin, hair and nails
13. conception (women/men) or maintaining pregnancy (women)
14. breastfeeding the baby
15. other, please specify:________________________________

On average, how often do you use/have you used a nutritional supplement based on omega-3 fatty acids:

1. every day
2. two or more times a week
3. once a week
4. once every two weeks
5. once a month

How long are you using/have you been using a dietary supplement based on omega-3 fatty acids:

1. less than 3 months
2. between 3 and 6 months
3. between 6 and 12 months
4. longer than 12 months

What dose of a dietary supplement based on omega-3 fatty acids do you use/have you used?

1. I take it roughly, I don't follow any special recommendations
2. the one recommended by the manufacturer (specified on the product declaration)
3. the one that was advised to me by the person who recommended me to use a dietary supplement based on omega-3 fatty acids
4. the one I found myself (in the literature, on the Internet, etc.)
5. other, please specify:___________________

On whose recommendation do you use/have you used these nutritional supplements?

1. I decided to use them myself
2. on the recommendation of a healthcare professional - doctor, pharmacist and/or dentist
3. on the recommendation of a close person - family member, partner or friend
4. I saw an advertisement on television/newspaper/internet
5. on the recommendation of a sports coach
6. other, please specify:_____________________

Have you informed your doctor that you use/have used nutritional supplements based on omega-3 fatty acids?

1. Yes
2. No

Where did you buy the dietary supplement based on omega-3 fatty acids (*it is possible to circle more than one answer*):

1. in the pharmacy
2. in a drug store (e.g. Lilly drugstore, DM...)
3. in general stores (supermarkets, markets...)
4. in health food stores
5. online
6. in sports supplement stores (vitamins and dietary supplements store)
7. other, specify:_____________________

Based on which criteria did you choose a dietary supplement based on omega-3 fatty acids (*it is possible to circle more than one answer*):

1. I bought the one that was recommended to me
2. I bought the one for which I have information that is considered or has been shown to be of good quality
3. I bought the one that I know was produced by a good and well-known manufacturer
4. I bought the one that suits me in terms of price
5. I bought the one with attractive packaging
6. other, specify:_______________________

knowledge and attitudes

On a scale of 1 to 5, rate how much you agree with the following statements:

	I completely disagree	I disagree	I am not sure	I agree	I completely agree
Omega-3 fatty acids are natural.	1	2	3	4	5
Omega-3 fatty acids are safe (no adverse health effects).	1	2	3	4	5
Omega-3 fatty acids are effective.	1	2	3	4	5
They can be used indefinitely.	1	2	3	4	5
They can be used in unlimited quantities.	1	2	3	4	5
They can be given to babies and children.	1	2	3	4	5
They can be given to the elderly population.	1	2	3	4	5
They can be given to pregnant and lactating women.	1	2	3	4	5
They can be used together with other medicines.	1	2	3	4	5
They can be used at any time of the day, regardless of the meal schedule.	1	2	3	4	5

Name the omega-3 fatty acid(s) you know: (*open answer, not mandatory*)

____

Name the natural sources (raw materials) from which omega-3 fatty acids are obtained: (*open answer, not mandatory*)

____

IF THE ANSWER IS NO

Why don't you use omega-3 fatty acid supplements? (*it is possible to circle more than one answer*)

1. I don't think I need them
2. I am not interested in their use
3. I think that I get everything I need with my usual diet
4. I don’t like to take medicines and nutritional supplements, except when absolutely necessary
5. I am not convinced of the effectiveness of the dietary supplement
6. People close to me (family, friends, colleagues, ...) think that it is not necessary to use these nutritional supplements
7. religious reasons

8. they are not suitable for people who are vegan or vegetarian
9. I think their price is too high
10. I have no particular reason
11. I don't have enough information about these supplements
12. I have not considered the use of these supplements
13. I am irresponsible when taking supplements (I don't have a habit of taking supplements)
14. other, specify:_______________

In which case(s) would you still consider the use of dietary supplements based on omega-3 fatty acids? (*it is possible to circle more than one answer*)

1. I would not consider
2. if these nutritional supplements were recommended to me by a healthcare professional - doctor, pharmacist, dentist
3. if these nutritional supplements were recommended to me by a close person - a family member, partner or friend
4. if I saw a convincing advertisement on television/newspaper/internet promoting these nutritional supplements
5. if I have an illness or impaired health condition in which the application is justified
6. if I had a confirmation of the safety of the application
7. if I had confirmation of the effectiveness of the application
8. if the price were lower
9. if I had more information about supplements based on omega 3 fatty acids
10. other, specify:________________________________

DIET

Do you apply/have you applied any special type of diet:

YES NO

Indicate what type of diet it is: (*it is possible to circle more than one answer*)

1. vegetarian or vegan diet
2. nutrition with organic food
3. raw food diet
4. diet with an increased amount of fruits and vegetables, and a reduced amount of meat
5. gluten-free diet
6. diet adapted to diabetes and insulin resistance
7. diet adapted to elevated blood fats
8. diet adapted to lactose intolerance (milk and milk products)
9. sugar-free diet ("keto diet")
10. other, please specify: ________________________
11. other types of diet that are not included in the previous answers (chrono diet, autoimmune protocol, candida diet...)

Do you eat fish?

YES NO

What type of fish do you eat most often?

1. fatty fish (salmon, herring, mackerel, tuna, carp, "blue fish")
2. lean fish (hake, cod, sole, toothfish, sardine, sea bream, perch, "white fish")
3. both fatty and lean, equally

How often do you eat fish?

1. every day
2. two or more times a week
3. once a week
4. once every two weeks
5. once a month

Do you eat other seafood?

YES NO

What other seafood do you eat most often (*it is possible to circle more than one answer*):

1. squid
2. octopuses
3. shellfish (mussels, oysters,.....)
4. caviar
5. crabs, lobsters...
6. other, please specify:_____________________

Do you eat any of the listed types of food (*it is possible to circle more than one answer*):

1. nuts (hazelnuts, walnuts, almonds...)
2. linseed
3. hemp seed
4. chia seeds
5. soybean
6. edamame (unripe soybeans)
7. none of the above

Which cooking oil/oils/fat do you most often use in your diet (*it is possible to circle more than one answer):*

1. animal fat
2. sunflower oil
3. olive oil
4. linseed oil
5. grape seed oil
6. coconut oil
7. pumpkin oil
8. sesame oil
9. palm oil
10. rapeseed oil
11. butter
12. other, please specify:____________

Do you use any of the listed oils: (*it is possible to circle more than one answer*)

1. evening primrose oil
2. borage oil
3. safflower oil
4. hemp oil
5. pomegranate oil
6. raspberry seed oil
7. black currant seed oil
8. apricot kernel oil
9. black cumin oil
10. none of the above

APPLICATION IN BABIES AND CHILDREN

Do you have children?

YES NO

How many children you have? ___________________

Are you a parent of a baby or a child under 3?

YES NO

Do you give/have you given any nutritional supplement based on omega-3 fatty acids to a baby or child up to 3 years of age?

YES NO

Please state the name(s) of nutritional supplements based on omega-3 fatty acids that you give/have given to a baby or child up to 3 years of age:

____

State the reason why do you give/have you been giving a baby or a child up to 3 years of age a nutritional supplement based on omega-3 fatty acids (*it is possible to circle more than one answer*):

1. I don't have a particular reason, I believe they are good for general health
2. due to insufficient intake through the usual diet
3. to improve the health of the heart and blood vessels
4. for the brain and intellectual functions
5. to improve the state of immunity
6. against allergies and/or respiratory diseases
7. other, specify:_______________________________

On average, how often do you give/have you given a baby or child up to 3 years old a nutritional supplement based on omega-3 fatty acids:

1. every day
2. two or more times a week
3. once a week
4. once every two weeks
5. once a month

How long do you give/have you been giving your baby or child up to 3 years old a dietary supplement based on omega-3 fatty acids:

1. continuously (regularly)
2. less than 3 months

What dose of dietary supplement based on omega-3 fatty acids do you give/have you given to a baby or child up to 3 years old?

1. roughly, I don't follow any specific recommendations
2. the one recommended by the manufacturer (listed on the product declaration)
3. the one that was advised to me by the person who recommended me to use a dietary supplement based on omega-3 fatty acids
4. the one I found myself (in the literature, on the Internet, etc.)
5. other, please specify:_____________________

On whose recommendation do you give/have you given these nutritional supplements to a baby or a child up to 3 years old?

1. I decided by myself
2. on the general recommendation of a healthcare professional - doctor, pharmacist and/or dentist
3. on the specific recommendation of a pediatrician
4. on the recommendation of a close person - family member, partner or friend
5. I saw a convincing advertisement on television/newspaper/internet
6. other, specify:______________________

Have you informed the pediatrician that you are/have been giving your child nutritional supplements based on omega-3 fatty acids?

1. Yes
2. No

knowledge and attitudes

On a scale of 1 to 5, rate how much you agree with the following statements:

	I completely disagree	I disagree	I am not sure	I agree	I completely agree
They could be given to babies and children up to 3 years of age.	1	2	3	4	5
They should be given to babies and children up to 3 years of age.	1	2	3	4	5
They could be given to babies and children from 3 to 7 years of age.	1	2	3	4	5
They should be given to children from 3 to 7 years of age.	1	2	3	4	5
They could be given to children from 7 to 12 years of age.	1	2	3	4	5
They should be given to children from 7 to 12 years of age.	1	2	3	4	5

Is your child on a special type of diet:

YES NO

Indicate what type of diet it is: (*it is possible to circle more than one answer*)

1. vegetarian or vegan diet
2. gluten-free diet
3. diet adapted to diabetes
4. diet adapted to lactose intolerance (milk and milk products)
5. nutrition of babies/children prone to stomach problems (intestinal colic)
6. nutrition of babies/children prone to vomiting due to regurgitation of stomach contents
7. other, specify: ________________________

Does your child eat fish?

YES NO

What type of fish does your child eat most often?

1. fatty fish (salmon, herring, mackerel, tuna, carp, "blue fish")
2. lean fish (hake, cod, sole, toothfish, sardine, sea bream, perch, "white fish")
3. both fatty and lean, equally

How often does your child eat fish?

1. every day
2. two or more times a week
3. once a week
4. once every two weeks
5. once a month

Does your child eat any of the listed types of food (*it is possible to circle more than one answer*):

1. nuts (hazelnuts, walnuts, almonds...)
2. linseed
3. hemp seed
4. chia seeds
5. soybean
6. edamame (unripe soybeans)
7. none of the above

Which edible oil/oils/fat do you most often use in your child's diet (*it is possible to circle more than one answer*)?

1. animal fat
2. sunflower oil
3. olive oil
4. linseed oil
5. grape seed oil
6. coconut oil
7. pumpkin oil

8. sesame oil
9. palm oil
10. rapeseed oil
11. none of the above
12. other, please specify:____________

APPLICATION IN PREGNANT AND BREASTFEEDING WOMEN

Note: the questions in this segment are only for women

(*respondents stating in the first (socio-demographic) part that they are male, and still answering this segment, will be deleted in data processing*)

Are you currently pregnant?

YES NO

Which trimester are you in currently?

1. first (0-3 months)
2. second (4-6 months)
3. third (7-9 months)

Did you use nutritional supplements based on omega-3 fatty acids before pregnancy?

YES NO

Is pregnancy the main reason for current use of omega-3 fatty acid supplements?

1. Yes, that is the most important reason
2. It is one of the more important reasons
3. No, it is not the reason for using these dietary supplements
4. I do not use these dietary supplements

Is the recommendation of your gynecologist the main reason for your use of dietary supplements based on omega-3 fatty acids?

1. Yes
2. It is one of the more important reasons
3. No
4. I do not use these dietary supplements

On a scale of 1 to 5, rate how much you agree with the following statements about omega-3 fatty acid supplements:

	I completely disagree	I disagree	I am not sure	I agree	I completely agree
They could be given to pregnant women.	1	2	3	4	5
They should be given to pregnant women.	1	2	3	4	5

Are you <u>currently</u> breastfeeding?

YES NO

How long have you been breastfeeding (in months)?

____

Is breastfeeding the main reason for current use of omega-3 fatty acid supplements?

1. Yes, that is the most important reason
2. It is one of the more important reasons
3. No, it is not the reason for using these dietary supplements
4. I do not use these dietary supplements

Is the recommendation of your healthcare professional (eg gynecologist or visiting nurse) the main reason for your use of dietary supplements based on omega-3 fatty acids?

1. Yes, it is the main reason
2. It is one of the more important reasons
3. No, it is not the reason for using these dietary supplements
4. I do not use these nutritional supplements

On a scale of 1 to 5, rate how much you agree with the following statements about omega-3 fatty acid supplements:

	I completely disagree	I disagree	I am not sure	I agree	I completely agree
They could be given to women who are breastfeeding.	1	2	3	4	5
They should be given to women who are breastfeeding.	1	2	3	4	5
